# Candidate SNP Markers of Gender-Biased Autoimmune Complications of Monogenic Diseases Are Predicted by a Significant Change in the Affinity of TATA-Binding Protein for Human Gene Promoters

**DOI:** 10.3389/fimmu.2016.00130

**Published:** 2016-04-04

**Authors:** Mikhail P. Ponomarenko, Olga Arkova, Dmitry Rasskazov, Petr Ponomarenko, Ludmila Savinkova, Nikolay Kolchanov

**Affiliations:** ^1^Institute of Cytology and Genetics, Siberian Branch of Russian Academy of Sciences, Novosibirsk, Russia; ^2^Novosibirsk State University, Novosibirsk, Russia; ^3^Children’s Hospital Los Angeles, Los Angeles, CA, USA

**Keywords:** gene, promoter, TATA-binding protein, TBP-binding site, single nucleotide polymorphism, expression change, gender-biased autoimmune disease, SNP marker

## Abstract

Some variations of human genome [for example, single nucleotide polymorphisms (SNPs)] are markers of hereditary diseases and drug responses. Analysis of them can help to improve treatment. Computer-based analysis of millions of SNPs in the 1000 Genomes project makes a search for SNP markers more targeted. Here, we combined two computer-based approaches: DNA sequence analysis and keyword search in databases. In the binding sites for TATA-binding protein (TBP) in human gene promoters, we found candidate SNP markers of gender-biased autoimmune diseases, including rs1143627 [cachexia in rheumatoid arthritis (double prevalence among women)]; rs11557611 [demyelinating diseases (thrice more prevalent among young white women than among non-white individuals)]; rs17231520 and rs569033466 [both: atherosclerosis comorbid with related diseases (double prevalence among women)]; rs563763767 [Hughes syndrome-related thrombosis (lethal during pregnancy)]; rs2814778 [autoimmune diseases (excluding multiple sclerosis and rheumatoid arthritis) underlying hypergammaglobulinemia in women]; rs72661131 and rs562962093 (both: preterm delivery in pregnant diabetic women); and rs35518301, rs34166473, rs34500389, rs33981098, rs33980857, rs397509430, rs34598529, rs33931746, rs281864525, and rs63750953 (all: autoimmune diseases underlying hypergammaglobulinemia in women). Validation of these predicted candidate SNP markers using the clinical standards may advance personalized medicine.

## Introduction

Recent studies (1) showed that the imbalance between effectors and regulators of immune responses causes autoimmune diseases. Self-antigen tolerance characterizes a healthy immune system, whereas impairment of this tolerance leads to autoimmune diseases. Changes in the expression of self-antigens in response to inflammation, tissue lesions, viruses, free radicals, radiation, and pharmaceuticals may trigger autoimmune pathogenesis (2, 3). According to the concept of molecular mimicry, antibodies induced by an infection attack self-antigens that are similar to the pathogen’s epitopes. This concept is a common explanation for the development of autoimmune diseases, i.e., destruction of host tissues by the host immune system (4, 5). More than 100 autoimmune diseases are already known, among them, the 8 most prevalent disorders are psoriasis, rheumatoid arthritis, type I diabetes mellitus, multiple sclerosis, systemic lupus erythematosus, Crohn’s disease, ulcerative colitis, and systemic scleroderma (6). Genotyping of geographic subpopulations (7, 8) revealed genetic diversity of immune responses and the single nucleotide polymorphisms (SNPs) involved (9). Women have greater immune responsiveness than men do, and it manifests itself in the fourfold prevalence of autoimmune diseases among women (10). Sex hormones amplify this hyperimmune response as do adolescence, pregnancy, and menopause stress-related hormonal status of women (11–14).

Overall, sex differences in immune capabilities and autoimmune diseases are an established fact, and precise clinical observations in patients and experiments on animal models underlie the mainstream scientific research aimed at elucidating these phenomena in clinical practice. The gender-biased interactions between microorganisms and the human host (15, 16); the effects of diets (16), sex hormones (17), and the X chromosome inactivation (18) on the immune response; and activities of regulatory genes located on the X chromosome (19) are hot topics in this field of research. In addition to this mainstream research into gender-biased autoimmune diseases, the nascent postgenomic predictive preventive personalized medicine (20) offers hope of elucidation of the pathogenesis of such diseases. To this end, it would be worthwhile to use SNP markers of autoimmune diseases as additional genome-wide informative landmarks. This way, a physician may analyze these SNP markers in his/her patients to improve treatment; in addition, the patients can modify their lifestyle accordingly to reduce the risk of autoimmune complications of their illnesses. We conducted the present study in accordance with this new auxiliary strategy as an adjunctive treatment for prevention of autoimmune complications of monogenic diseases. For example, this kind of adjunctive modality improves survival in metastatic breast cancer (21).

Postgenomic SNP identification is a part of the 1000 Genomes project (22), whose results are available in the dbSNP database (23). The UCSC Genome Browser (24) visualizes the human reference genome (25, 26) as the ancestral variant for all SNPs. It allows clinical researchers to choose an appropriate set of SNPs for genotyping of patients in comparison with healthy volunteers in order to identify/validate disease-related SNP markers (27). Furthermore, these researchers can estimate the population frequencies (28), genetic drifts (29), expressivity, and penetrance (30) of these markers. The data from these clinical studies are available in many databases (31–34) designed for postgenomic predictive preventive personalized medicine (20).

Computer-based analysis of hundreds of millions of unannotated SNPs can make the search for SNP markers more targeted and less expensive (35). To this end, bioinformatics researchers (36–52) rate SNPs using genome-wide maps of genes, functional sites, nucleosomes, interchromosomal contacts, chromatin immunoprecipitation (ChIP) data, and transcriptomes in health (53), in disease (54), and after treatment (55). The Central Limit Theorem ensures an increase in the accuracy of these estimates with the increasing number, diversity, representativeness, and completeness of genome-wide maps (56). Due to this approach, thousands of SNP markers have been found within protein-coding regions of genes (32) [where SNPs alter gene products (57)] but only a few SNP markers among millions of SNPs in regulatory regions of genes (23, 25). The majority of the regulatory SNP markers are located in the [−70; −20] region relative to the transcription start site (58), where TATA-binding protein (TBP) binds to DNA (59). Among ~2600 human DNA-binding proteins (60), TBP is among the most important ones: a knockout (61) or knockdown (62) of the *TBP* gene is lethal because RNA polymerase II binds to the TBP–DNA complex to induce formation of the transcription preinitiation complex (58). Many experiments have shown that an increase in TBP’s affinity for the promoter of a gene manifests itself in overexpression of this gene and *vice versa* (63–65). Finally, data on high-throughput sequencing of immunoprecipitated chromatin (ChIP-Seq) validated the TBP-binding sites in most genes in yeast (66) and in mice (67). Similarly, *in silico* estimates that were verified by *in vivo* bioluminescence validated TBP-binding sites in humans (68).

Earlier, we developed a computer-based statistical estimate of SNP-caused alteration of TBP’s binding affinity for promoters (69); this estimate can predict a change in expression of the human genes associated with monogenic diseases (70). Then, we empirically verified such predictions using an electrophoretic mobility shift assay (EMSA) under equilibrium (71) and non-equilibrium (72) conditions *in vitro* as well as in real-time mode (73). Next, we conducted a comparison of these predictions with independent experimental data published by various authors (74–77). Finally, we developed the Web service SNP_TATA_Comparator^1^ (78) and showed how to use it in practice (79).

Recently, we expanded the applicability of our Web service (79) from the known SNP markers of monogenic diseases to candidate SNP markers of obesity-related complications of monogenic diseases (80). Here, we continued this extension in relation to autoimmune complications of monogenic diseases, and this work is expected to advance postgenomic predictive preventive personalized medicine (20).

## Materials and Methods

### DNA Sequences

**Figure 1:**
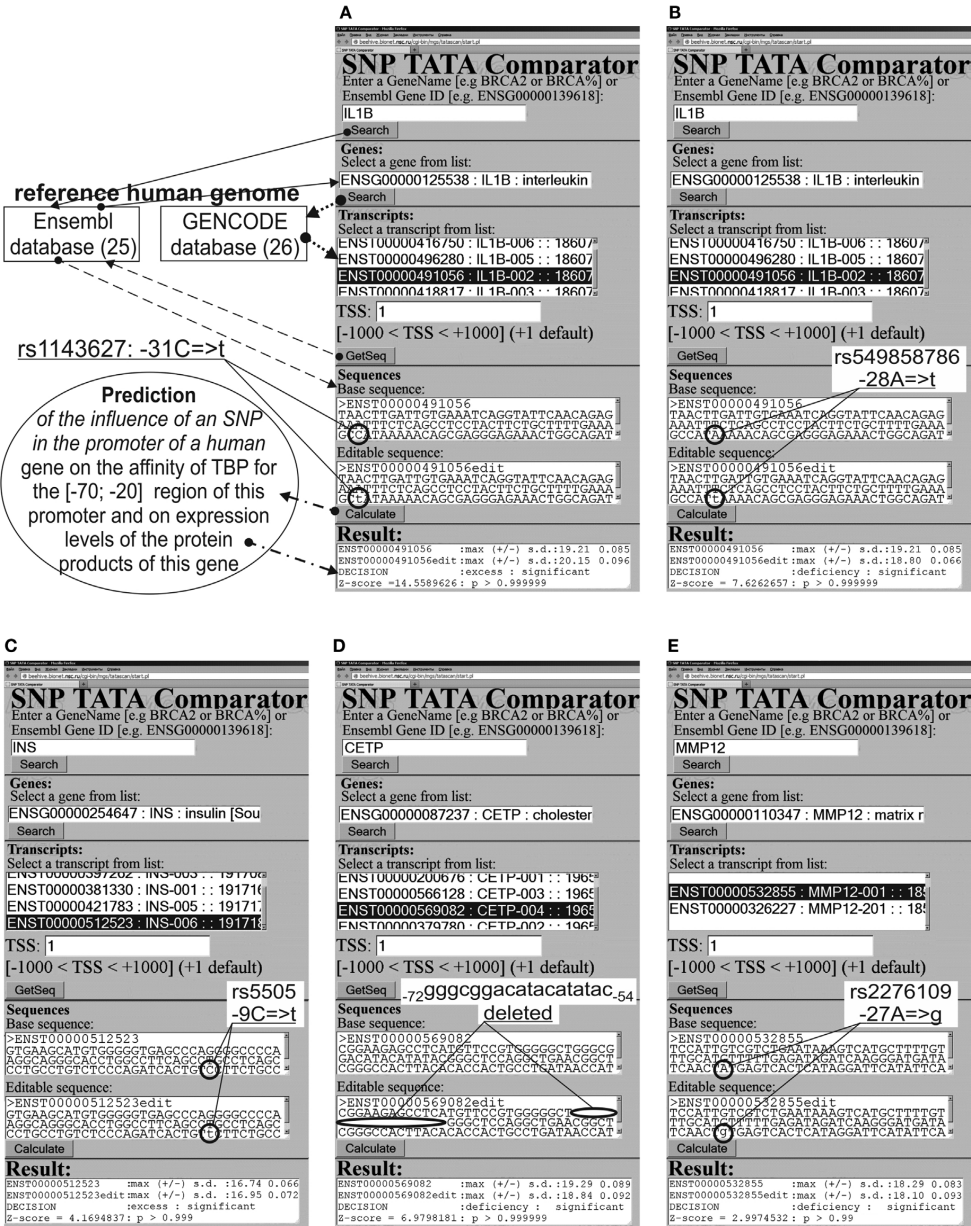
**Examples of the predictions by SNP_TATA_Comparator (79) for statistically significant alterations in the expression of human genes**. **(A,C–E)** Known biomedical SNP markers of autoimmune diseases; **(B)** the candidate SNP marker near the known SNP marker rs1143627 [see **(A)**].

We analyzed 90-bp DNA sequences {s_−90_ … s_−1_} of the proximal regions of core promoters in ancestral and minor variants (hereinafter: wt and mut variants, respectively) of the human genes from the default version of the reference human genome (where s_0_ is the transcription start site; s_i_ ∈ {a, c, g, t}); here, we used the current major assembly release GRCh38(NCBI)/hg38(UCSC) [in the terms used by the UCSC Genome Browser (24)]. Figure 1 shows examples of the ancestral (text box “Base sequence”) and minor variants (text box “Editable sequence”) of several biomedical and nearby candidate SNP markers in the promoters of some human genes. Arrows illustrate the process of retrieval of the ancestral DNA from Ensembl (25) on the basis of the list of transcripts for the reference human genome in GENCODE (26). The minor variants were compiled manually by introducing substitutions, deletions, and/or insertions into the ancestral variant.

One can find a brief description of our computer-based method for DNA sequence analysis (81–91) in Supplementary Material and a detailed description in our recent article (79).

### Keyword Search

Here, we are expanding the range of applicability of our freely available Web-service (79) from monogenic diseases only to their autoimmune complications. For this purpose, we try to emulate the successful real-life expansion of diagnostic capabilities of the well-known SNP marker rs1143627 (Table 1: the first row). One can see that this marker of Graves’ disease (92, 93) was discovered in association with gastric cancer (94); after that, it was implicated in hepatocellular carcinoma (95), next in excess adiposity in older men (96), and then in non-small cell lung cancer (97), major recurrent depression (98), gastric ulcer, and chronic gastritis (99). Accordingly, we combined sequence analysis and keyword searches.

**Table 1:** **Known and nearby candidate SNP markers (of autoimmune diseases) that can change affinity of TBP for a human gene promoter**.

Gene (OMIM ID)	dbSNP (12) rel. 142 or see (Ref)	5′ flank $\frac{\text{wt}}{\text{mut}}$ 3′ flank	*K*_D_, nM				Known diseases and observations in the case of known SNP markers (Ref) *or hypothetical ones in the case of the candidate SNP markers predicted by us in this work; see* Figure 2	(Ref) or (this work)
			$\frac{\text{wt}}{\text{mut}}$	Δ	*Z*	α		
*IL1B* (147720)	rs1143627	ttttgaaagc $\frac{\text{c}}{t}$ ataaaaacag	$\frac{5}{2}$	↑	15	10^−6^	Graves’ disease whose risk is higher in females with skewed X chromosome inactivation	(92, 93, 94–100) (this work) (101–105)
							Recurrent major depression; chronic gastritis; gastric ulcer; gastric, liver, and non–small cell lung cancers; greater body fat;	
							*Hypothetically, cachexia in rheumatoid arthritis (double prevalence among women)*	
	rs549858786	tgaaagccat $\frac{\text{a}}{t}$ aaaacagcga	$\frac{5}{7}$	↓	8	10^−6^	*Hypothetically, rheumatoid arthritis (double prevalence among women)*	(this work) (101–105)
*INS* (176730)	rs5505	agatcactgt $\frac{\text{c}}{t}$ cttctgccat	$\frac{53}{44}$	↑	4	10^−3^	Type 1 diabetes after neonatal diabetes mellitus (women who had 6q24-transient neonatal diabetes mellitus are at risk of a relapse)	(31, 106–108)
	rs563207167	tcagccctgc $\frac{\text{c}}{t}$ tgtctcccag	$\frac{53}{44}$	↑	4	10^−3^	*Hypothetically, type 1 diabetes after neonatal diabetes mellitus (women who had 6q24-transient neonatal diabetes mellitus are at risk of a relapse)*	(this work)
	rs11557611	gatcactgtc $\frac{\text{c}}{t}$ ttctgccatg	$\frac{53}{60}$	↓	2	0.05	*Hypothetically, demyelinating diseases (thrice more prevalent among young white women than among non-white individuals)*	(this work) (11, 109, 110)
*CETP* (118470)	See Ref. (111)	cgtgggggct $\frac{\text{18 bp}}{-}$ gggctccagg	$\frac{4}{7}$	↓	7	10^−6^	Hyperalphalipoproteinemia that reduces atherosclerosis risk and corresponds to coronary artery disease risk that is twice lower in women than in men	(111–113)
	rs17231520	ggggctgggc $\frac{\text{g}}{a}$ gacatacata	$\frac{4}{2}$	↑	10	10^−6^	*Hypothetically, hypoalphalipoproteinemia that causes atherosclerosis, atherosclerosis-related autoimmune and coronary artery diseases (double prevalence among women)*	(this work) (111–115)
	rs569033466	atacatatac $\frac{\text{g}}{a}$ ggctccaggc	$\frac{4}{3}$	↑	4	10^−3^		
*MMP12* (601046)	rs2276109	gatatcaact $\frac{\text{a}}{\text{g}}$ tgagtcactc	$\frac{11}{14}$	↓	3	10^−2^	Low risk of asthma and systemic sclerosis exacerbated by menopause in women	(116–118) (this work) (12, 119–121)
							*Hypothetically, low risk of psoriasis that is associated with increased risk of cardiovascular diseases with age in women*	
	rs572527200	gatgatatca $\frac{\text{a}}{\text{g}}$ ctatgagtca	$\frac{11}{14}$	↓	3	10^−2^	*Hypothetically, low risk of systemic sclerosis exacerbated by menopause in women; psoriasis associated with increased risk of cardiovascular diseases with age in women; asthma*	(this work)

*wt, ancestral allele; mut, minor allele; *K*_D_, an estimate (79) of the dissociation constant *K*_D_ of the TBP-promoter complex corresponding to the conditions *in vitro* (71); Δ, a change: overexpression (↑), deficiency (↓), norm (=); *Z*, *Z*-score; α = 1 − *p*, significance [where *p* is the probability rate (79) shown in Figure 1]; TF, transcription factor; EMSA, electrophoretic mobility shift assay*.

**Figure 2:**
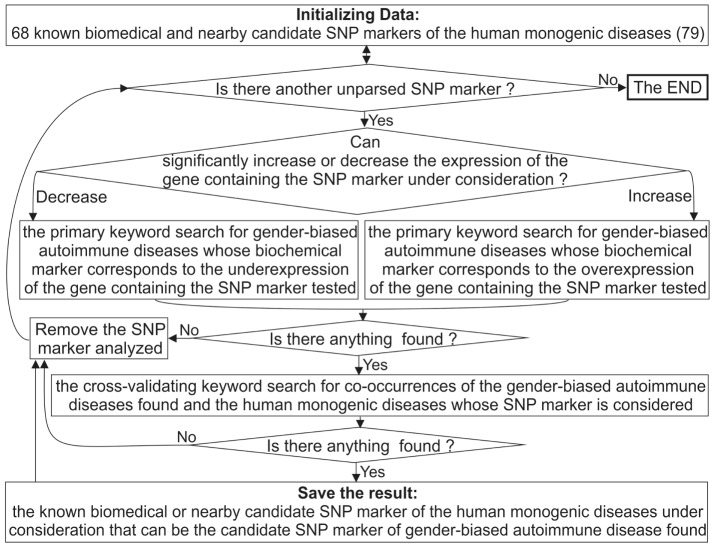
**A flow chart showing extension of the diagnostic potential of 68 known and candidate SNP markers (79) from monogenic diseases to gender-biased autoimmune diseases**.

Figure 2 depicts a flow chart of our extension of the diagnostic potential of 68 known and candidate SNP markers (79) from monogenic diseases to gender-biased autoimmune diseases. To this end, for each SNP marker causing either significant overexpression or underexpression of the human gene containing this SNP, we manually performed a keyword search using proper combinations of the terms “overexpression,” “deficiency,” “knockout,” “women,” and many terms corresponding to various autoimmune diseases in public databases, as described in detail elsewhere (100). If we successfully found the autoimmune diseases whose biochemical marker corresponds to the expression change of the gene containing the SNP marker in question, then we did one more keyword search for co-occurrences of the found autoimmune diseases and the monogenic diseases whose SNP marker was being analyzed. This additional keyword search can serve as cross-validation of sorts at the level of a rough qualitative estimate without strict statistical criteria.

Our heuristic interpretation of our predicted cases of significant overexpression or underexpression of the human genes is shown in italics in the second rightmost column of Tables 1–3, with the word *“hypothetically”* in front of these interpretations. These are clinical data found during our manual keyword search, with the corresponding references in the rightmost column of these tables [also shown in italics and marked with the phrase “*(this work)*”].

## Results and Discussion

### The Results on the Known SNP Markers of Gender-Biased Autoimmune Diseases

Table 1 shows the applicability of our Web service (79) to analysis of gender-biased autoimmune diseases. Let us consider only one example from these results in detail in order to briefly describe all the other results from Tables 1–3 in a similar way.

#### The Human *IL1B* Gene

The human *IL1B* gene (interleukin 1β) in its promoter contains a known SNP marker of intractable Graves’ disease (rs1143627) (92). This pathology has the highest prevalence among females with skewed X chromosome inactivation (93). This SNP is a substitution of a minor T for an ancestral C at position −31 (hereafter denoted as −31C → T) in the promoter of this gene. It converts a non-canonical variant of the TBP-binding site in DNA, C_−31_ATAAAA, to the canonical TATA box: T_−31_ATAAAA. In case of the minor allele −31T, the estimate of TBP’s affinity for the *IL1B* promoter (see “Materials and Methods”: Supplementary Material, Eqs. 1–4), equaling 20.15 ± 0.10 ln-units (2 nM, according to Table 1), is significantly stronger (*Z* = 14.56, α < 10^−6^) than the affinity corresponding to the ancestral allele (−31C, 19.21 ± 0.09 ln-units; 5 nM). This significant increase in affinity of TBP for the minor variant of the *IL1B* promoter corresponds (63–65) to overexpression of this gene (designated as “↑” in Table 1). This prediction is consistent with clinical studies showing overexpression of *IL1B* in patients with Graves’ disease (92).

Because clinical records of the patients with these diseases confirmed *IL1B* overexpression, we performed a primary keyword search for “*IL1B* overexpression” as a biochemical marker of “gender-biased autoimmune diseases” in various databases (hereafter, see Figure 2). The last column of Table 1 shows the result obtained. One study (101) showed an association of the biochemical marker “*IL1B* overexpression” with cachexia as a complication of rheumatoid arthritis (double prevalence among women). Therefore, we propose rs1143627 as a candidate SNP marker for this pathology (Table 1).

The 90-bp promoter that we studied here contains the candidate SNP marker rs549858786 reported in our recent work (79). This SNP can significantly decrease TBP’s affinity for the *IL1B* promoter (Figure 1B) and cause underexpression of *IL1B*. A primary keyword search yielded laboratory data (102) on a mouse model of human rheumatoid arthritis; these data showed that an *IL1B* deficiency elevates the risk of this autoimmune disease [its risk is twofold higher among women than among men (101)]. The final cross-validating keyword search (hereinafter: see Figure 2) yielded a retrospective study (103) showing significantly frequent co-occurrence of some pairs of rheumatic diseases and cancers. In addition, another research group (104) reported that rheumatoid arthritis can be a complication of gastric disease treatment that is based on non-steroidal anti-inflammatory drugs. One more paper (105) revealed that a high body-mass index is associated with a reduced risk of rheumatoid arthritis in men but not in women. These three independent findings mostly support our prediction of two rheumatoid arthritis-related candidate SNP rs1143627 and rs549858786 markers.

#### The Human *INS* Gene

The human *INS* gene (insulin) contains the known SNP marker of type 1 diabetes after neonatal diabetes mellitus (rs5505) (106); this mutation can increase the blood level of insulin (Figure 1C), promote the development of type 1 diabetes (107), and increase the risk of other autoimmune diseases (Table 1). In addition, it was reported that women who have 6q24-transient neonatal diabetes mellitus are at risk of a relapse (108). We predicted (79) insulin overexpression in the case of the unannotated SNP rs563207167 in the same promoter, as is the case for rs5505 (Table 1). Thus, we propose rs563207167 as a candidate SNP marker of the same gender-biased autoimmune disease (106–108).

Regarding another unannotated SNP rs11557611, we predicted (79) an insulin deficiency (Table 1), and we found (using the primary keyword search) a medical hypothesis that *in vivo* insulin deficiency is a possible cause of demyelinating disease (109), thrice more prevalent among young white women than among non-white individuals (11). The cross-validating keyword search pinpointed clinical cases of demyelinating diseases as a complication of type 1 diabetes in children (110). Thus, we propose rs11557611 as a candidate SNP marker of autoimmune demyelinating diseases.

#### The Human *CETP* Gene

The human *CETP* gene (plasma cholesteryl ester transfer protein) contains a minor variant of the promoter: the deletion G_–72_GGCGGACATACATATAC_–54_ between positions −72 and −54, which was implicated in hyperalphalipoproteinemia that reduces atherosclerosis risk (111). This effect is twofold greater in women than in men (112). This SNP significantly lowers expression of *CETP* (Figure 1D). Regarding two candidate SNP markers – rs17231520 and rs569033466 (located within the above-mentioned 18-bp deletion between positions −72 and −54) – we predicted (79) significant overexpression of *CETP* (Table 1) and linked them (by means of a primary keyword search) with hypoalphalipoproteinemia that increases the risk of premature atherosclerosis-related coronary disease and autoimmune diseases (113, 114). The cross-validating keyword search produced an article on atherosclerosis as a risk factor of coronary diseases (115). This finding may serve as a rationale for our prediction of rs17231520 and rs569033466 as candidate SNP markers of both atherosclerosis-related autoimmune disease and coronary diseases.

#### The Human *MMP12* Gene

The human *MMP12* gene (matrix metallopeptidase 12) contains the known SNP marker (rs2276109) of a reduced risk of chronic asthma in children and in smokers (116, 117) as well as with the reduced risk of systemic sclerosis (117) that is exacerbated by menopause in women (12). This SNP causes *MMP12* underexpression (Figure 1E), in agreement with clinical data (118). A primary keyword search produced empirical data (119) that overexpression of this gene in human keratinocytes may be associated with psoriasis (12). After that, the cross-validating keyword search showed that asthma elevates the risk of psoriasis (120), whereas psoriasis and systemic sclerosis often co-occur (121). We also predicted another candidate SNP marker (rs572527200) (79), whose numerical values were identical to those in the case of the above-mentioned rs2276109. Thus, we propose rs2276109 and rs572527200 as candidate SNP markers of a low risk of psoriasis, asthma, and systemic sclerosis.

### The Results on the Known SNP Markers of Monogenic Diseases That May Also Be Candidate SNP Markers of Gender-Biased Autoimmune Diseases

#### The Human *HBB* and *HBD* Genes

The human *HBB* and *HBD* genes (β- and δ-chains of hemoglobin, respectively) contain seven known SNP markers (rs34500389, rs33981098, rs33980857, rs34598529, rs33931746, rs397509430, and rs35518301) of resistance to malaria and thalassemia (Cooley’s anemia) (122). These SNPs cause underexpression of these genes (122) (Table 2). In addition, we predicted three candidate SNP markers (rs281864525, rs63750953, and rs34166473) of the same disorders (79) because these SNPs can also cause underexpression of *HBB* and *HBD* (Table 2). A primary keyword search revealed a retrospective analysis of autoimmune disease cases in children (123), where anemia is associated with a high risk of autoimmune diseases underlying hypergammaglobulinemia in women. One more cross-validating keyword search produced a review (124) showing an association between thalassemia and autoimmune diseases. Thus, we predicted ten candidate SNP markers of a gender-biased autoimmune complication of hypergammaglobulinemia (rs35518301, rs34166473, rs34500389, rs33981098, rs33980857, rs397509430, rs34598529, rs33931746, rs281864525, and rs63750953) (123).

**Table 2:** **Known and nearby candidate SNP markers (of anemias) that may be candidate SNP markers of underlying autoimmune diseases in women with hypergammaglobulinemia**.

Gene (OMIM ID)	dbSNP (12) rel. 142 or see (Ref)	5′ flank $\frac{\text{wt}}{\text{mut}}$ 3′ flank	*K*_D_, nM				Known diseases and observations in the case of known SNP markers (Ref) *or hypothetical ones in the case of the candidate SNP markers predicted by us in this work; see* Figure 2	(Ref) or (this work)
			$\frac{\text{wt}}{\text{mut}}$	Δ	*Z*	α		
*HBD* (142000)	rs35518301	caggaccagc $\frac{\text{a}}{g}$ taaaaggcag	$\frac{4}{8}$	↓	11	10^−6^	Resistance to malaria and Cooley’s anemia (δ-thalassemia) *that can hypothetically increase the risk of an autoimmune disease in women with hypergammaglobulinemia*	(122) (this work) (123, 124)
	rs34166473	aggaccagca $\frac{\text{t}}{c}$ aaaaggcagg	$\frac{4}{8}$	↓	18	10^−6^	*Hypothetically, Cooley’s anemia with high risk of autoimmune diseases underlying hypergammaglobulinemia in female patients*	(this work)
*HBB* (141900)	rs34500389	cagggctggg $\frac{\text{c}}{a,t,g}$ ataaaagtca	$\frac{5}{6}$	↓	3	10^−2^	Malaria resistance, Cooley’s anemia (β-thalassemia);	(122) (this work) (123)
	rs33981098	agggctgggc $\frac{\text{a}}{g,c}$ taaaagtcag	$\frac{5}{9}$	↓	10	10^−6^	*hypothetically, high risk of autoimmune diseases in women with hypergammaglobulinemia*	
	rs33980857	gggctgggca $\frac{\text{t}}{a,g,c}$ atacaacagt	$\frac{5}{21}$	↓	27	10^−6^		
	rs397509430	gggctgggca $\frac{\text{t}}{-}$ atacaacagt	$\frac{5}{29}$	↓	34	10^−6^		
	rs34598529	ggctgggcat $\frac{\text{a}}{g}$ aaagtcaggg	$\frac{5}{18}$	↓	24	10^−6^		
	rs33931746	gctgggcata $\frac{\text{a}}{g,c}$ aagtcagggc	$\frac{5}{11}$	↓	14	10^−6^		
	rs281864525	tgggcataaa $\frac{\text{a}}{c}$ gtcagggcag	$\frac{5}{7}$	↓	7	10^−6^	*Hypothetically, malaria resistance, Cooley’s anemia with high risk of autoimmune diseases in women with hypergammaglobulinemia*	(this work)
	rs63750953	ctgggcataa $\frac{\text{aa}}{-}$ gtcagggcag	$\frac{5}{8}$	↓	9	10^−6^		
*ACKR1* (613665)	rs2814778	ttggctctta $\frac{\text{t}}{c}$ cttggaagca	$\frac{10}{12}$	↓	4	10^−3^	Resistance to malaria, low white-blood-cell count (anemia), asthma, high total IgE levels, and reduced neutrophil count;	(125–128) (this work) (123, 129–131)
							*Hypothetically, autoimmune diseases (excluding multiple sclerosis and rheumatoid arthritis) underlying hypergammaglobulinemia in women*	

*wt, ancestral allele; mut, minor allele; *K*_D_, an estimate (79) of the dissociation constant *K*_D_ of the TBP-promoter complex corresponding to the conditions *in vitro* (71); Δ, a change: overexpression (↑), deficiency (↓), norm (=); *Z*, *Z*-score; α = 1 − *p*, significance [where *p* is the probability rate (79) shown in Figure 1]; TF, transcription factor; EMSA, electrophoretic mobility shift assay*.

#### The Human *ACKR1* Gene

The human *ACKR1* gene (atypical chemokine receptor 1) contains the known SNP rs2814778 marker of malaria resistance (125) and of a lower white-blood cell count (126), a reduced neutrophil count (127), asthma, and high total IgE levels (128). This SNP can reduce the expression of this gene (79), in line with other studies (125–128), as shown in Table 2. A primary keyword search allowed us to propose rs2814778 as a candidate SNP marker of a lower risk of multiple sclerosis (129) and rheumatoid arthritis (130) and a candidate marker of a higher risk of other autoimmune diseases underlying hypergammaglobulinemia in women (123), as shown in Table 2. Indeed, the final cross-validating keyword search uncovered a retrospective association between asthma and subsequent autoimmune diseases diagnosed at least 5 years after asthma (131).

#### The Human *StAR* Gene

The human *StAR* gene (steroidogenic acute regulatory protein) contains a biomedical SNP marker of hypertension in diabetes (rs16887226) (132), with the highest risk at the waist circumference >87 cm in women and >99 cm in men (133). The EMSA showed that this SNP disrupts a tissue-specific unknown transcription factor-binding site rather than the ubiquitous TBP-binding site (132) and reduces this gene’s expression. Table 3 shows that our prediction (79) is supported by these EMSA data (132). Near this known rs16887226 marker, we predicted a candidate SNP marker of hypertension in diabetes (rs544850971) (79) because it can damage the TBP-binding site and thus reduce *StAR* expression, as rs16887226 does. Using a primary keywords search, we further predicted that during a deficiency in *StAR* as a mediator between the circadian and immune systems, both rs16887226 and rs544850971 can serve as separate candidate SNP markers of low resistance to endotoxins (134) and of a good chance for partial restoration of this resistance by training in postmenopausal women (135). Lastly, the cross-validating keyword search pinpointed a clinical association (136) between the endothelial dysfunction and the hypertension, diabetes, and endotoxemia pathologies whose candidate SNP rs16887226 and rs544850971 markers were predicted here (Table 3).

**Table 3:** **Known and nearby candidate SNP markers (of monogenic diseases) that may also be candidate SNP markers of autoimmune diseases**.

Gene (OMIM ID)	dbSNP (12) rel. 142 or see (Ref)	5′ flank $\frac{\text{wt}}{\text{mut}}$ 3′ flank	*K*_D_, nM				Known diseases and observations in the case of known SNP markers (Ref) *or hypothetical ones in the case of the candidate SNP markers predicted by us in (this work); see* Figure 2	(Ref) or (this work)
			$\frac{\text{wt}}{\text{mut}}$	Δ	*Z*	α		
*StAR* (600617)	rs16887226	cagccttcag $\frac{\text{c}}{t}$ gggggacatt	$\frac{10}{10}$	=	0	0.5	Hypertensive diabetic patients (EMSA: an unknown TF-binding site is disrupted rather than a TBP-binding site)	(132) (this work) (133–136)
							*Hypothetically, low resistance to endotoxins (diet and training may restore this resistance in obese postmenopausal women)*	
	rs544850971	tcagcggggg $\frac{\text{a}}{g}$ catttaagac	$\frac{10}{12}$	↓	5	10^−2^	*Hypothetically, hypertension in diabetes (waist circumference* ≥*87 cm in women) and low resistance to endotoxins (diet training may restore this resistance in obese postmenopausal women)*	(this work)
*APOA1* (107680)	(137)	tgcagacata $\frac{\text{a}}{c}$ Ataggccctg	$\frac{3}{4}$	↓	5	10^−6^	Hematuria; fatty liver; obesity	(137) (this work) (11, 113, 114, 138)
							*Hypothetically, hypoalphalipoproteinemia causes atherosclerosis-related autoimmune diseases (double prevalence among women)*	
*F3* (134390)	rs563763767	ccctttatag $\frac{\text{c}}{t}$ gcgcggggca	$\frac{3}{2}$	↑	6	10^−6^	Myocardial infarction; thrombosis	(139) (this work) (14, 140, 141)
							*Hypothetically, Hughes syndrome-associated thrombosis (lethal during pregnancy)*	
*TNFRSF18* (603905)	rs111426889	gtgctataaa $\frac{\text{c}}{t}$ gccgccccct	$\frac{4}{2}$	↑	8	10^−6^	Resistance to parasites	(142) (this work) (11, 143, 144)
							*Hypothetically, some autoimmune diseases (fourfold prevalence among women)*	
*NOS2* (163730)	(147)	gtataaatac $\frac{\text{t}}{c}$ tcttggctgc	$\frac{2}{1}$	↑	3	10^−2^	Resistance to malaria or epilepsy	(145–147) (this work) (148–150)
							*Hypothetically, inflammation and tissue damage in pemphigus vulgaris (double prevalence among women)*	
*MBL2* (154545)	rs72661131	tctatttcta $\frac{\text{t}}{c}$ atagcctgca	$\frac{2}{4}$	↓	12	10^−6^	Variable immunodeficiency; preeclampsia; and stroke	(151–153) (this work) (13, 154–160)
							*Hypothetically, preterm delivery in pregnant diabetic women and cardiovascular events in rheumatoid arthritis*	
	rs562962093	atctatttct $\frac{\text{a}}{g}$ tatagcctgc	$\frac{2}{5}$	↓	15	10^−6^	*Hypothetically, high risk of preterm delivery in pregnant diabetic women, cardiovascular events in rheumatoid arthritis*	(this work)
	rs567653539	tttctatata $\frac{\text{g}}{a}$ cctgcaccca	$\frac{2}{1}$	↑	12	10^−6^	*Hypothetically, high risk of cardiovascular events in rheumatoid arthritis*	(this work)
*DHFR* (126060)	rs10168	ctgcacaaat $\frac{\text{a}}{g}$ gggacgaggg	$\frac{15}{9}$	↑	9	10^−6^	Resistance to methotrexate treatment of leukemia	(161) (this work) (162–165)
							*Hypothetically, resistance to methotrexate in autoimmune diseases, without negative effects on bone mineral density in women*	
*SOD1* (147450)	rs7277748	ggtctggcct $\frac{\text{a}}{g}$ taaagtagtc	$\frac{2}{7}$	↓	17	10^−6^	Amyotrophic lateral sclerosis (double prevalence among men), *which may hypothetically be an autoimmune disease and, in addition, autoimmune diseases can often precede amyotrophic lateral sclerosis*	(166) (this work) (167–169)

*wt, ancestral allele; mut, minor allele; *K*_D_, an estimate (79) of the dissociation constant *K*_D_ of the TBP-promoter complex corresponding to the conditions *in vitro* (71); Δ, a change: overexpression (↑), deficiency (↓), norm (=); *Z*, *Z*-score; α = 1 − *p*, significance [where *p* is the probability rate (79) shown in Figure 1]; TF, transcription factor; EMSA, electrophoretic mobility shift assay*.

#### The Human *APOA1* Gene

The human *APOA1* gene (apolipoprotein A-I) contains the −35A → C substitution inside a proven TATA box (the canonical form of the TBP-binding sites). This substitution reduces the expression of this gene and thus is the SNP marker of hematuria, fatty liver, and obesity (137). A primary keyword search revealed a knockout APOA1^−/−^ mouse model of human hypoalphalipoproteinemia (113) characterized by an elevated risk of atherosclerosis-related autoimmune diseases (118) [double prevalence among females (112)]. After that, the cross-validating keyword search yielded a review showing obesity-induced development of atherosclerosis in children and in adolescents (138). Thus, we predicted this known SNP marker of obesity to be a candidate SNP marker of atherosclerosis-related autoimmune diseases (Table 3).

#### The Human *F3* Gene

The human *F3* gene (coagulation factor F3) contains the known SNP rs563763767 marker of the high risk of myocardial infarction and thromboembolism whose molecular cause is *F3* overexpression (139) as we predicted *in silico* and confirmed in our experiments *in vitro* (72). A primary keyword search revealed that *F3* overexpression is a biochemical marker of Hughes syndrome-associated thrombosis (140), which is lethal during pregnancy (14). The cross-validating keyword search produced a clinical practice report on Hughes syndrome as an earlier easily detectable and preventable cause of myocardial ischemia (141). Thus, we predicted (Table 3) the known SNP marker of myocardial infarction (rs563763767) to be a candidate SNP marker of Hughes syndrome whose early detection is easy and can prevent (141) Hughes syndrome-associated thrombosis (140), which is lethal during pregnancy (14).

#### The Human *TNFRSF18* Gene

The human *TNFRSF18* gene (glucocorticoid-induced TNFR-related protein) contains the known SNP marker of resistance to parasites (rs111426889) (142) due to overexpression of this gene (79). A primary keyword search yielded a minireview (143) showing that *TNFRSF18* overexpression can cause development of some autoimmune diseases with fourfold prevalence among women (10). Then, the cross-validating keyword search produced laboratory data showing that diabetic mice are resistant to mycobacteria, whereas a mycobacterial infection prevents this autoimmune disease (144). Thus, we predicted that the known SNP marker of resistance to parasites (rs111426889) can additionally be a candidate SNP marker of autoimmune diseases (Table 3).

#### The Human *NOS2* Gene

The human *NOS2* gene (inducible nitric oxide synthase 2) contains the −51T → C substitution as a known SNP marker of epilepsy (145) and resistance to malaria (146, 147) due to overexpression of this gene (79) (Table 3). A primary keyword search pointed to an empirical study on a mouse model of human pemphigus vulgaris (148) where *NOS2* overexpression as a biochemical marker was found to be associated with inflammation and tissue damage as two complications of this autoimmune disease (148). This disease is twofold more prevalent among women than among men (149). Using the cross-validating keyword search, we found a clinical case report of pemphigus vulgaris after antiepileptic therapy (150). On this basis, we predicted that this known SNP marker of epilepsy can be a candidate SNP marker of inflammation and tissue damage as complications of pemphigus vulgaris (Table 3).

#### The Human *MBL2* Gene

The human *MBL2* gene (soluble mannose-binding lectin 2) contains a known SNP marker (rs72661131) of variable immunodeficiency (151), preeclampsia (152), and stroke (153). This SNP impairs expression of this gene, as we predicted (79) and proved in experiments under both equilibrium (71) and non-equilibrium (72) conditions *in vitro*. A primary keyword search produced clinical findings of a high risk of preterm delivery in pregnant diabetic women (13) and a report about cardiovascular events in rheumatoid arthritis (154); the latter is twice more frequent in women than in men (101). Near this SNP rs72661131, we found two unannotated SNPs (rs562962093 and rs567653539), which can cause the MBL2 underexpression and overexpression, respectively (Table 3). The cross-validating keyword search yielded six articles (155–160) showing that the variable immunodeficiency, preeclampsia, stroke disorders, and autoimmune diseases are clinically associated. Thus, we predicted three candidate SNP markers of preterm delivery in pregnant diabetic women (13) and cardiovascular events in rheumatoid arthritis (154) (rs72661131, rs562962093, and rs567653539; Table 3).

#### The Human *DHFR* Gene

The human *DHFR* gene (dihydrofolate reductase) contains the known SNP marker of resistance to methotrexate treatment in children with acute lymphoblastic leukemia (rs10168) (161). This SNP causes overexpression of DHFR (79). A primary keyword search pointed to autoimmune diseases that are commonly treated with this drug (162) because it has no negative effects on bone mineral density in women (163). Next, the cross-validating keyword search produced two clinical reports (164, 165) showing that autoimmune diseases elevate the risk of leukemia. These data favor our prediction that the known SNP marker of resistance to methotrexate treatment in leukemia (rs10168) can additionally be a candidate SNP marker of the same drug resistance in autoimmune diseases (162, 163).

#### The Human *SOD1* Gene

The human *SOD1* gene (soluble superoxide dismutase 1) contains the known SNP marker of familial amyotrophic lateral sclerosis (rs1143627) (166) caused by underexpression of this gene, as we predicted *in silico* (79) and proved in *in vitro* experiments (72). Although this degenerative disorder of the central nervous system is not generally considered an autoimmune disease, our primary keyword search revealed a relevant empirical study on a mouse model of human multiple sclerosis (167). It shows an association of amyotrophic lateral sclerosis with autoimmune diseases (167). It is worth mentioning that amyotrophic lateral sclerosis occurs twice as often in men (168); this situation is not characteristic of autoimmune diseases. The cross-validating keyword search yielded an epidemiologic review (169) of the autoimmune diseases preceding amyotrophic lateral sclerosis. Thus, we predicted the known SNP marker of amyotrophic lateral sclerosis (rs1143627) to be a candidate SNP marker of autoimmune diseases.

### The Results of the Statistical Comparison between the Computationally Predicted and Experimentally Measured –ln(*K*_D_) Values of the TBP-Promoter Affinity

**Figure 3:**
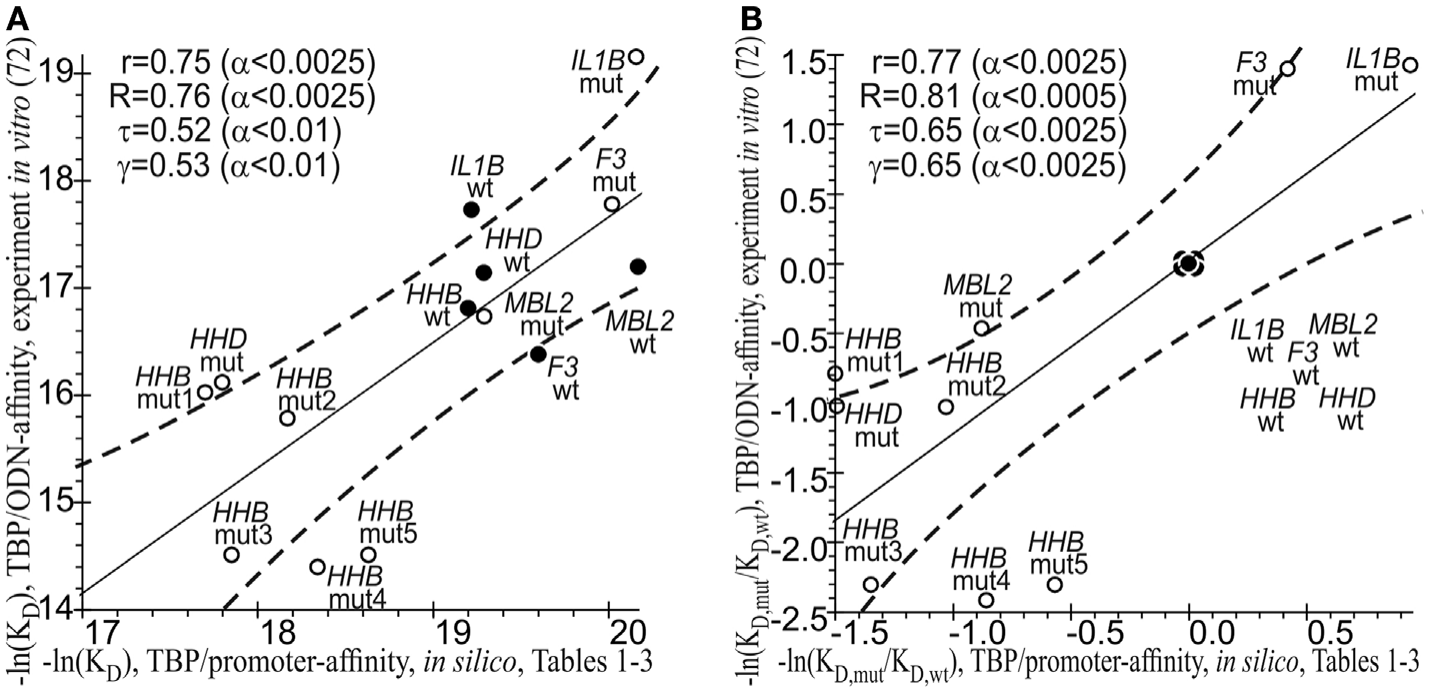
**The significant correlations between the −ln(*K*_D_) values predicted (Tables 1–3, the *x*-axis) and measured by an electrophoretic mobility shift assay (EMSA) *in vitro* (the *y*-axis) from our previous article (72). (A)** Absolute scales; **(B)** relative scales. Solid and dashed lines denote the linear regression and boundaries of its 95% confidence interval, respectively; ● and ○ are the ancestral and minor alleles, respectively; *r*, *R*, τ, γ, and α are coefficients of Pearson’s linear correlation, Spearman’s rank correlation, Kendall’s rank correlation, and Goodman–Kruskal’s generalized correlation and their significance, respectively; mut1 = rs33980857:a, mut2 = rs33980857:c, mut3 = rs34598529:g, mut4 = rs3393174:g, and mut5 = rs3393174:c; ODN, oligodeoxyribonucleotide.

As a final cross-validation test, we conducted a statistical comparison between the −ln(*K*_D_) values of the TBP-promoter affinity that were predicted *in silico* (Tables 1–3) and measured by the EMSA *in vitro* (72). On an absolute natural logarithmic scale, Figure 3A shows a significant correlation, namely: linear (*r* = 0.75; α < 0.0025), Goodman–Kruskal’s generalized (γ = 0.53; α < 0.01), Spearman’s rank correlation (*R* = 0.76, α < 0.0025), and Kendall’s rank correlation (τ = 0.52; α < 0.01). On the other hand, Figure 3B shows this correlation on a relative natural logarithmic scale: *r* = 0.77 (α < 0.0025), γ = 0.65 (α < 0.0025), *R* = 0.81 (α < 0.0025), and τ = 0.65 (α < 0.0025). Thus, eight statistical tests indicated the robustness of the correlation between our predicted values (Tables 1–3) and empirical −ln(*K*_D_) values (72). This robustness can cross-validate our predictions that known and candidate SNP markers of monogenic diseases can be candidate SNP markers of autoimmune complications of these diseases.

### How to Use Candidate SNP Markers of Autoimmune Complications of Monogenic Diseases

This study is focused on the TBP-binding site because it is the best-studied site upstream of the transcriptional start of any mRNA in the genome, where RNA polymerase II binds to the anchoring TBP–DNA complex at the stage of preinitiation complex formation (58). As continuation of our previous analysis of monogenic diseases (74–79), here, in addition to the genetic susceptibility to diseases – previously the only known SNP manifestation – we identified associations with complications of autoimmune diseases (e.g., rs1143627: autoimmune diseases often precede amyotrophic lateral sclerosis), gender specificity of such complications (e.g., rs72661131 and rs562962093: preterm delivery in pregnant diabetic women), autoimmune complications of non-autoimmune diseases (e.g., Table 2: autoimmune diseases underlying hypergammaglobulinemia in women), gender-biased epigenetic regulation of gene expression (e.g., rs1143627: skewed X chromosome inactivation), drug resistance (e.g., rs10168: resistance to methotrexate treatment of autoimmune diseases), and the effects of a lifestyle in women (e.g., rs16887226 and rs544850971: diet and training can restore resistance to endotoxins). These findings extend the field of practical applications of our Web service due to the keyword searches (100).

Be that as it may, known SNP markers of monogenic diseases are known to cause these disease, whereas the questions “What is the cause?” and “What is the consequence?” in relation to the pathogenesis of autoimmune diseases are still the focus of active biomedical research (15–19). Each candidate SNP marker predicted here is only a genome-wide informative landmark in a patient with the minor allele of this SNP; this situation can help the patient and his/her doctor to improve the lifestyle and treatment, respectively, to prevent autoimmune complications of the illness in question. As an example, here we predicted a candidate SNP marker of Hughes syndrome-associated thrombosis (rs563763767), which is lethal during pregnancy (140), whereas Hughes syndrome is easy to diagnose early and is a preventable cause of myocardial ischemia (141). Keeping this additional information in mind, a pregnant woman with the minor allele of this SNP and her physician can arrange additional diagnostic tests to monitor emergence and development of the symptoms of the relevant autoimmune complications, in addition to an adjunctive treatment during her pregnancy. Similarly, parents of the obese children or adolescents with the –35C allele of the *APOA1* gene, when obesity was caused by their accelerated development and maturation, can modify the diet and lifestyle of their children to reduce the excess body fat before an imbalance of the immune system causes atherosclerosis. Moreover, two candidate SNP markers predicted by us (rs16887226 and rs544850971) would be interesting to obese postmenopausal women with the minor alleles of these SNPs who developed low resistance to endotoxins; these women can resort to training and dietary changes in order to restore this resistance (135). By the same token, all the other candidate SNP markers predicted here (Tables 1–3) may help both patients and clinicians to improve quality of life and efficiency of health care.

With this auxiliary bioinformatic approach, here we could perform only something like cross-validation with rough qualitative estimates and limitations of a keyword search in databases without exact statistical tests. Consequently, biomedical standardization of the SNP-disease association data available today (100) may advance postgenomic predictive preventive personalized medicine (20).

It should be noted that there are known problems with the computational prediction of the TBP-binding site because this site may shift depending on whether TBP interacts with an ancestral or minor allele of a human gene promoter (170). To address this problem, instead of computational prediction of the exact location of this 15-bp site within human gene promoters, we estimated the maximal value of TBP’s binding affinity for the whole 50-bp region where TBP binds to DNA of these promoters (59). In addition to the commonly accepted prediction criterion of the TBP-binding site [i.e., Bucher’s position-weight matrix score (86)], we took into account both prior and subsequent molecular events, such as TBP’s sliding along DNA (83) and stabilization of the TBP-promoter complex by bending of the DNA axis at a right angle (87), respectively (see Materials and Methods: Supplementary Material). In Figure 3, one can see the statistically significant correlations between our estimates *in silico* (this work) and empirical *in vitro* values (72) of TBP’s binding affinity for the human gene promoters. Moreover, these correlations are robust, i.e., they persist despite variations of linear, rank, or generalized correlation criteria. This robustness supports our results on the candidate SNP markers of autoimmune complications of monogenic diseases.

Finally, it is worth noting that our analysis of the candidate SNP markers of autoimmune diseases (Tables 1–3) will merely inform physicians about the degree of the molecular (e.g., *K*_D_ values, *Z*-score, and α value) and biomedical evidence (two rightmost columns in Tables 1–3) as a rationale for expensive and labor-consuming validation of a particular SNP in a particular disease. The decisive proof would be the significantly higher frequency in patients than in healthy people, and this frequency can be confounded by climate, environmental conditions, lifestyles, and the ethnic, social, age, and gender composition of cohorts (171). Because statistical significance of the predicted SNP markers varied from high confidence (α < 10^−6^) to borderline significance (α < 0.05), the proposed markers should be tested according to proper biomedical standards and protocols prior to application to clinical practice. For the best targeting of our analysis, we arranged the ancestral and minor alleles of each candidate SNP marker of autoimmune diseases by *K*_D_ values expressed as affinity of TBP’s binding to synthetic aptamers of double-stranded DNA 26 bp long, as we predicted for *in vitro* conditions (71). We found that these *K*_D_ values vary from 1 to 60 nM, whereas their variation among alleles of a certain SNP is within 1 nM, which is less than 2% of the *K*_D_ range. Thus, the allelic variation is too small for accurate experimental determination of differences in *K*_D_ without consideration of additional data on the expected range of the values to be measured. That is why the predicted *K*_D_ values (Tables 1–3) require empirical verification with sophisticated equipment (71–73).

## Conclusion

Here we predicted candidate SNP markers of gender-biased autoimmune complications of monogenic diseases (Tables 1–3). They are located within TBP-binding sites of human gene promoters. Validation of these markers in accordance with clinical standards can bridge the gap between the best-studied SNPs (within protein-coding regions of genes) and the worst-studied SNPs (in regulatory regions of genes). After that, the validated SNP markers can allow physicians to select the best treatment for each patient and may help patients to choose a lifestyle reducing the risk of autoimmune complications.

## Author Contributions

MP, manuscript writing; LS, manuscript editing; DR, software development and application; OA, data compilation; PP, data analysis; and NK, conceived of and supervised the study.

## Conflict of Interest Statement

The authors declare that the research was conducted in the absence of any commercial or financial relationships that could be construed as a potential conflict of interest.

## Footnotes

^1^http://beehive.bionet.nsc.ru/cgi-bin/mgs/tatascan/start.pl
