# Supplementary material for: Candidate SNP Markers of Gender-Biased Autoimmune Complications of Monogenic Diseases Are Predicted by a Significant Change in the Affinity of TATA-Binding Protein for Human Gene Promoters
Source: Front Immunol. 2016 Apr 4;7:130. doi: 10.3389/fimmu.2016.00130 (PMC4819121; doi:10.3389/fimmu.2016.00130)
Supplement: Supplementary file 1 [file Presentation_1.PDF]

Supplementary Material

Candidate SNP markers of gender-biased autoimmune complications of monogenic diseases are predicted by a significant change in the affinity of TATA-binding protein for human gene promoters

Mikhail Ponomarenko*, Olga Arkova, Dmitry Rasskazov, Petr Ponomarenko, Ludmila Savinkova, and Nikolay Kolchanov

*** Correspondence:** Mikhail Ponomarenko: pon@bionet.nsc.ru

1 A supplementary method for DNA sequence analysis

Each proximal 90-bp DNA sequence $\{s_{-90}\ldots s_{-1}\}$ of a given gene promoter (where $s_i \in \{a, c, g, t\}$; s_0 is the transcription start site) was characterized (the "Calculate" button and the "Result" text box in Figure 1) with the maximum value of $-\ln(K_D) \pm \delta$ among all estimates of TBP's binding affinity for the 26-bp window $\{s_{i-13}\ldots s_i\ldots s_{i+12}\}$ centered by the i-th position ranging from –70 to –20 for both DNA chains (79). Here, K_D is the equilibrium dissociation constant of the TBP-DNA complex expressed in moles per liter, M, and calculated by the linear-additive approximation of the three-step molecular mechanism of TBP binding to a promoter that was first predicted *in silico* (69), and, a year later, was discovered in an experiment *in vitro* (81). The K_D value was assessed by means of our empirical equation:

$$\boldsymbol{ln(K_D) = 10.9 - 0.2\{ln(K_{SLIDE}([TA]_{3'HALF}; minor\ groove\ width\ of\ B - helical\ DNA)) + ln(K_{STOP}(PWM_{TATA-box})) + ln(K_{BEND}([WR]_{FLANKS}; [TV]_{CENTER}))\};} \quad (1)$$

where 10.9 (natural logarithm units, ln-units) is the experimental nonspecific TBP-DNA affinity, 10^{-5} M (82); 0.2 = (15/26)/3 is the stoichiometric coefficient, the ratio of the empirically identified minimal length of a TBP-binding site (83) to the length of the sliding DNA window used that is additionally normalized to three steps of the TBP–DNA binding (69, 81). K_{SLIDE} is used for empirical evaluation of the equilibrium constant at step I (83) and denotes the mean specific affinity of TBP for double-stranded DNA. This affinity is calculated from the mean width of the minor DNA groove (84) at the center of the sliding DNA window and from the proportion of TA dinucleotides in the 3′ half of the sliding DNA window (85). K_{STOP} represents empirical evaluation of the equilibrium constant at step II and is calculated from Bucher's position-weighted matrix (PWM) as the commonly accepted criterion of the TATA box—the canonical variant of the TBP-binding site (86)—for the moment when TBP stops after encountering the TBP-binding site. K_{BEND} represents empirical evaluation of the equilibrium constant at step III: stabilization of the TBP–DNA complex by denaturation of the double-stranded DNA contacting TBP, resulting in bending of the axis of the DNA helix by an angle of 19° to 90° (87). In other words, this is the mean specific affinity of TBP for each of the single-stranded DNAs deduced from the proportion of TA-like dinucleotides WR = {TA, AA, TG, AG} and TV = {TA, TC, TG} (88) [notation according to the IUPAC-IUB nomenclature (89)] in the range of the maximum score of Bucher's PWM (86). All equations for K_{SLIDE}, K_{STOP}, and K_{BEND} were repeatedly published in our earlier works (69, 70, 74-80) with detailed descriptions. It was necessary for the present study to derive Eq. 1 (69) from our own experimental results documented in our ACTIVITY database (90).

Eq. 1 was proven by the data from 68 independent experiments [for a review, see (65)] and was verified by *in vitro* experiments under equilibrium (71), nonequilibrium (72), and real-time (73) conditions *in vitro*, with measurement of K_D values for complexes of TBP with 26-bp synthetic DNA aptamers containing natural TATA boxes of the human genes affected by the biomedical SNP markers.

We calculated the standard deviation of K_D estimates according to Eq. 1 for all the 3 × 26 = 78 possible mononucleotide substitutions within the 26-bp DNA sliding window from the value of the analyzed variant (i.e., ancestral or minor) of the DNA sequence under study, δ, as follows:

$$\boldsymbol{\delta = \frac{1}{78} \sum_{\varphi \epsilon \{a,t,g,c\}} \sum_{j=-13}^{12} ln\left(\frac{K_D(s_{i-13} \dots s_{i+j-1} s_{i+j} s_{i+j+1} \dots s_{-1})}{K_D(s_{i-13} \dots s_{i+j-1} \varphi s_{i+j+1} \dots s_{-1})}\right).} \quad (2)$$

Eq. 2 describes the measure of genetic stability of the K_D value of TBP's affinity for the TBP-binding site in a gene promoter, with respect to the minimal possible genomic changes: the mononucleotide substitutions within the core promoter in question (69, 70, 74-80).

Regarding the promoter DNA sequences of both minor (mut) and ancestral (wt) alleles of a given gene, we obtained the results of Eqs. 1 and 2, $\{-\ln(K_D^{(mut)}) \pm \delta_{(mut)}\}$ and $\{-\ln(K_D^{(wt)}) \pm \delta_{(wt)}\}$, and compared them using Fisher's Z-score:

$$\boldsymbol{Z = \frac{\left|ln\left(K_D^{(mut)}/K_D^{(wt)}\right)\right|}{\sqrt{\delta_{(mut)}^2 + \delta_{(wt)}^2}}.} \quad (3)$$

Eq. 3 was taken from the standard statistical package R (91) whose procedure transforms this Fisher's Z-score value (as input data) into the p value (as output data) of the probability rate of acceptance of the hypothesis "H_0: $K_D^{(mut)} \neq K_D^{(wt)}$" (where α = 1 – p corresponds to the statistical significance level).

Figure 1 shows (in the bottom line of the "Result" text box) the Z-score and p value, as do columns Z and α in Tables 1–3. Accordingly, we predicted the SNP-caused alteration in the expression of this gene using the following decision rule:

$$\begin{array}{c} \boldsymbol{IF\left\{-ln\left(K_D^{(mut)}\right)\ is\ statistically\ significantly\ greater\ than\ -ln\left(K_D^{(wt)}\right)\right\}} \\ \boldsymbol{THEN\{there\ is\ an\ excess\ of\ the\ allele\ mut\ of\ a\ given\ gene\ versus\ the\ allele\ wt\}} \\ \boldsymbol{ELSE\left[IF\left\{-ln\left(K_D^{(mut)}\right)\ is\ statistically\ significantly\ less\ than\ -ln\left(K_D^{(wt)}\right)\right\}\right.} \\ \boldsymbol{THEN\{there\ is\ a\ deficiency\ of\ the\ allele\ mut\ of\ a\ given\ gene\ versus\ the\ allele\ wt\}]} \\ \boldsymbol{OTHERWISE\{alteration\ of\ the\ expression\ of\ this\ gene\ is\ insignificant\}.} \end{array} \quad (4)$$

Eq. 4 means that significantly greater affinity of TBP for the minor variant than for the ancestral one corresponds to overexpression of the gene, and lower affinity corresponds to underexpression, as shown in numerous independent experiments (63–65). Examples of such predictions are shown in Figure 1 (the "Decision" line in the "Result" text box) and in the "Δ" column in Tables 1–3.
